# Sputum Eosinophil Level among Healthy Individuals Visiting Medicine Outpatient Department of a Tertiary Care Centre: A Descriptive Cross-sectional Study

**DOI:** 10.31729/jnma.7408

**Published:** 2022-03-31

**Authors:** Subash Pant, Prinsa Shrestha, Rajat Acharya, Pranita Gurung, Arpana Neopane

**Affiliations:** 1 Department of Internal Medicine, Kathmandu Medical College and Teaching Hospital, Sinamangal, Kathmandu, Nepal; 2 Kathmandu Medical College and Teaching Hospital, Sinamangal, Kathmandu, Nepal; 3 Department of Pathology, Kathmandu Medical College and Teaching Hospital, Sinamangal, Kathmandu, Nepal; 4 Department of Pulmonology, Sleep and Critical Care, Kathmandu Medical College and Teaching Hospital, Sinamangal, Kathmandu, Nepal

**Keywords:** *eosinophils*, *healthy volunteers*, *leukocyte count*, *reference values*, *sputum*

## Abstract

**Introduction:**

Sputum differentia! is the most comprehensive, and non-invasive investigation for evaluating airway inflammation because of its reliability, reproducibility and responsiveness. The interpretation of the results of induced sputum examination depends on knowledge of normal values from a healthy population. So far, the reference values of sputum differential cells in Nepalese population are not reported. Neutrophil and eosinophil are used to assess the inflammatory response of the airway. The aim of our study was to detect the eosinophil level in the sputum of healthy individuals with normal pulmonary function.

**Methods:**

A descriptive cross-sectional study was conducted in a tertiary care hospital, from November 1, 2021 to December 31, 2021 after taking ethical clearance from Institutional Review Board (Reference number: 1507202106). Convenience sampling was done. Collected data was entered and analysed using Statistical Package for the Social Sciences version 24.0. Point estimate at 95% was calculated along with mean and standard deviation for continuous data.

**Results:**

Among 139 induced sputum samples, 7 (5.03%) had eosinophils present in their sputum (1.40 to 8.67 at 95% Confidence Interval). The age range of the participants was 18 years to 79 years with mean age of 39.5 ± 15.06. Prevalence of eosinophilia (percentage of eosinophil >3) was 4 (2.88%).

**Conclusions:**

The prevalence of eosinophilia in our study was found to be similar to published literature. The results of the present study show that there is a paucity of eosinophils with predominance of neutrophils, macrophages and lymphocytes in induced sputum samples of healthy non-smoker adults with normal pulmonary function test.

## INTRODUCTION

Induced sputum is an useful investigation for evaluating the presence, type and degree of inflammation in the airways of lungs.^1^ It helps to determine various phenotypes of asthma,^2-4^ chronic bronchitis,^3^ and study the effect of treatment on these airway diseases.^5-7^ Sputum differential is the most comprehensive, noninvasive examination of airway inflammation because of it reliability, reproducibility and responsiveness.^8-10^ Neutrophil and eosinophil are used to assess the inflammatory response of airway.^1^

The interpretation of the results of induced sputum examination depends on knowledge of normal values from a healthy population but there have been only few such studies.^11-16^ So far, the reference values of sputum differential cells in healthy Nepalese population are not reported.

The aim of our study was to detect the eosinophil level and to find reference values of normal sputum differential cell percentages in the sputum of healthy individuals with normal pulmonary function tests.

## METHODS

A descriptive cross sectional study was done in Kathmandu Medical College and Teaching Hospital, Kathmandu, Nepal from November 1, 2021 to December 31, 2021. Ethical clearance was taken from the institutional review board of Kathmandu Medical College and Teaching Hospital with reference number: 1507202106.

Study population was individuals visiting the outpatient department of Internal Medicine of Kathmandu Medical College and Teaching Hospital. Inclusion criteria for participants in the study are:

1. Healthy non-smoker adult of age >18 years who provide consent for the study,
2. No history of any chronic respiratory conditions,
3. No history of respiratory infection within four weeks before the recruitment,
4. No history of contact with patients with respiratory illness within four weeks before the recruitment,
5. No history of atopy,
6. Individual with normal radiological findings in chest X-ray, and
7. Normal pulmonary function according to European Respiratory Society (ERS)/American Thoracic Society (ATS) guideline i.e. Forced expiratory volume in 1 second [FEV1] >80% predicted, and FEV1 to forced vital capacity [FVC] ratio >0.7.^17,18^

Convenience sampling was done and sample size was calculated using the formula:

n = (Z^2^ × p × q) / e^2^

  = (1.96^2^ × 0.5 × 0.5) / 0.085^2^

  = 133

Where,

n = required sample size
z = 1.96 at 95% Confidence Interval (CI)
p = prevalence taken as 50% for maximum sample size calculation
q = 1-p
e = margin of error, 8.5%

Hence, the total sample size was calculated to be 133. However, 139 samples were taken.

The participants who fulfilled the inclusion criteria underwent induced sputum collection with the help of inhalation of 3% hypertonic saline. The procedure was stopped if the subject experienced respiratory distress. Those with unsuccessful sputum collection or inadequate sputum after induction were excluded from the study. Treatment with inhaled bronchodilator was given to those subjects who developed respiratory distress during the procedure. Sputum sample was then preceded for examination of cytology. Sputum differential of neutrophil, macrophage, lymphocyte, and eosinophil was obtained from each sample in percentage. Eosinophilia is defined as ≥3% eosinophils in sputum.^19^

Statistical Package for the Social Sciences (SPSS) version 24.0 was used for data entry and analysis. The percentage of sputum differential cell counts were expressed as arithmetic mean±SD, median and interquartile range (IQR). The normal range was expressed by 10^th^ and 90^th^ percentiles. Point estimate at 95% Confidence Interval was calculated.

## RESULTS

Total 139 healthy adults who fulfilled the inclusion criteria were included in the study among which 7 (5.03%) had eosinophils present in their sputum (1.40 to 8.67 at 95% Confidence Interval). There were 88 (63.3%) male and 51 (36.7%) female. The age range of the participants was 18 years to 79 years with mean age of 39.50 ±15.06. The FEV1 / FVC among the participants were 0.83±0.05 (Table 1).

**Table 1:** Baseline characteristics of participants.

Characteristics	Mean±SD	Median (Interquartile range)
Age	39.50±15.06	36 (23)
Body Mass Index (BMI)	24.913.99	24.38 (6.27)
FEV1 (Litre)	3.11±2.13	2.96 (1.06)
FEV1 (% predicted)	104.96±16.25	103 (23)
FVC (Litre)	3.54±0.94	3.58 (1.34)
FVC (% predicted)	106.14±17.36	103 (21)
FEV1 /FVC	0.83±0.05	0.83 (0.08)

**Figure 1:**
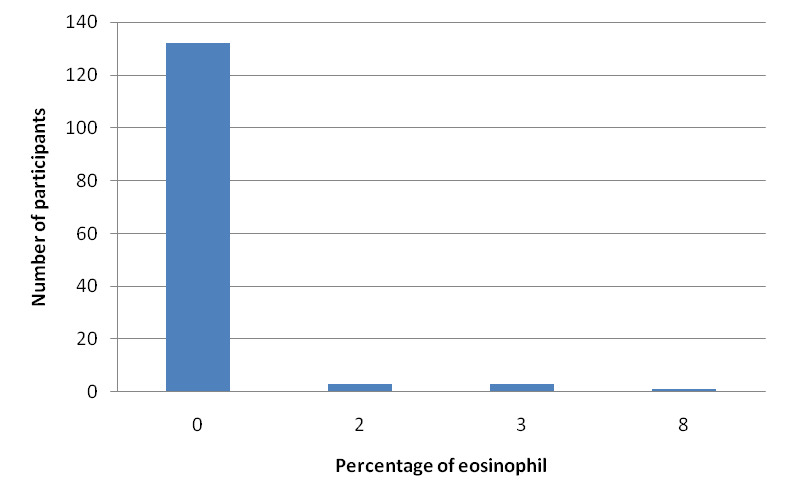
Eosinophil level in induced sputum samples (n= 139).

The eosinophil count among induced sputum samples was mostly zero percent in 132 (94.96%), two percent in 3 (2.16%), three percent in 3 (2.16%) and eight percent in 1 (0.72%) individuals (Figure 1). Prevalence of eosinophilia (percentage of eosinophil >3) was 4 (2.88%).

Neutrophils were predominant cells in induced sputum with a mean of 56.54±16.65% and median of 60% (Table 2).

**Table 2:** Induced sputum differential percentages.

Alveolar cells	Mean±SD	Median (IQR)	10th percentile	9th percentile
Macrophages	21.96±17.38	18 (17)	5	45
Neutrophils	56.54±16.65	60 (518)	30	75
Lymphocytes	21.36±11.48	20 (112)	8.8	34.3
Eosinophils	0.16±0.85	-	-	-

Aside from eosinophils, on sputum examination neutrophils were present in 139 (100%), macrophages in 127 (91.4%), lymphocytes in 139 (100%), and eosinophils in 7 (5%) individuals. (Table 3).

**Table 3:** Presence of different alveolar cell types among induced sputum samples according to age group.

Age group	Number of participants	No of participants n (%) with presence of			
		Neutrophils	Macrophages	Lymphocytes	Eosinophils
18 to 30	50	50 (100%)	47 (94%)	50 (100%)	2 (4%)
31 to 50	58	58 (100%)	51 (87.93%)	58 (100%)	4 (6.89%)
51 to 79	31	31 (100%)	29 (93.54%)	31 (100%)	1 (3.23%)

## DISCUSSION

In this study, we found the eosinophil level and normal sputum differential cell percentage in the sputum of healthy non-smoker adults visiting medicine OPD of KMCTH with normal pulmonary function test by studying the sputum characteristics of 139 participants who fulfilled the inclusion criteria. Compared with previous studies, this study includes a larger number of participants and a broad range of all ages (18 to 79 years); moreover, the numbers of subjects are comparable in all ages.

The mean values in our sample are in agreement with the findings with other studies with healthy subjects.^11-16^ The results of our study show that neutrophils predominate in sputum of healthy adults; macrophages and lymphocytes are less common, while eosinophils are seldom present which is similar to those in literature.

In our study, the percentage of eosinophils ranged from zero percent to 8%. The mean reference value of eosinophils was 0.16% which was comparable to studies conducted by Veras TN, et al. (0.1%),^16^ Belda J, et al. (0.4%),^11^ and Spanevello A, et al. (0.6%).^12^ The reference values of percentage of eosinophil in sputum are lower than 2.0% in most studies,^11,12,14,16^ but the values from Canada^13^ and Korea^15^ are above 2.5%, which may attribute to the inclusion of subjects with allergy history. Therefore, it is reasonable to define the abnormality of eosinophil counts if the percentage is above 2.5%, which might be suggestive of airway eosinophilic inflammatory conditions.

In our study, the proportion of neutrophils is 56.54%, which is higher than that reported in previous studies including Belda J, et al. (37.5%),^11^ Luo W, et al. (38.3%),^14^ Davidson WJ, et al. (50.3%),^13^ Spanevello A, et al. (27.3%),^12^ and Veras TN, et al. (23.4%).^16^ However, the percentage of lymphocytes in our study is 21.36% which is much higher than that reported in previous studies including Belda J, et al. (1.0%),^11^ Spanevello, et al. (1.0%),^12^ Davidson WJ, et al. (2.6%),^13^ and Veras TN ,et al. (3.1%).^16^ It suggests that reference values might vary by geographic location. In our study, the mean reference value of macrophages was 21.96% which is lower compared to previous studies conducted by Belda J, et al. (58.8%)," Luo W, et al. (58.9%)^14^, Spanevello A, et al. (69.2%),^12^ and Veras TN, et al. (68.4%).^16^

Limitation of our study was the way of recruitment. Participants were recruited from hospital visitors, which could not exclude the possibility of comorbidities other than asthma, respiratory diseases, or recent systemic infection. However, due to methodological considerations, sputum induction and processing should be performed with specialised instruments and very near to laboratories, making community based sampling difficult. Also, since our study was a singlecentred study, with convenience sampling, the results cannot be generalised to the whole population.

Another limitation of our study is that cut-off values of FEV_1_ and FEV_1_/FVC may vary according to age group. Since participants included in our study are of various age groups, fixed cut-off values of predicted FEV_1_ >80% and FEV_1_/FVC >0.70 defining normal pulmonary function may not hold true for all the participants.

## CONCLUSIONS

The prevalence of eosinophilia in our study was found to be similar to published literature. The present study is the first attempt to determine the eosinophil level of sputum differential counts in healthy Nepalese adults with a normal pulmonary function. The results of the present study show that there is a paucity of eosinophils with predominance of neutrophils, macrophages and lymphocytes in induced sputum samples of healthy nonsmoker individuals with normal pulmonary function. It is recommended to conduct a national multi-center study to establish the reference value of sputum cell counts for the Nepalese population.

## References

[1] Pin I, Gibson PG, Kolendowicz R, Girgis-Gabardo A, Denburg JA, Hargreave FE (1992). Use of induced sputum cell counts to investigate airway inflammation in asthma.. Thorax..

[2] Simpson JL, McElduff P, Gibson PG (2010). Assessment and reproducibility of non-eosinophilic asthma using induced sputum.. Respiration..

[3] Boorsma M, Lutter R, van de Pol MA, Out TA, Jansen HM, Jonkers RE (2007). Repeatability of inflammatory parameters in induced sputum of COPD patients.. COPD..

[4] Moritz P, Steidle LJ, Felisbino MB, Kleveston T, Pizzichini MM, Pizzichini E (2008). Determination of the inflammatory component of airway diseases by induced sputum cell counts: use in clinical practice.. J Bras Pneumol..

[5] Green RH, Brightling CE, Woltmann G, Parker D, Wardlaw AJ, Pavord ID (2002). Analysis of induced sputum in adults with asthma: identification of subgroup with isolated sputum neutrophilia and poor response to inhaled corticosteroids.. Thorax..

[6] Jayaram L, Pizzichini MM, Cook RJ, Boulet LP, Lemiere C, Pizzichini E (2006). Determining asthma treatment by monitoring sputum cell counts: effect on exacerbations.. Eur Respir J..

[7] Leigh R, Pizzichini MM, Morris MM, Maltais F, Hargreave FE, Pizzichini E (2006). Stable COPD: predicting benefit from high-dose inhaled corticosteroid treatment.. Eur Respir J..

[8] Pizzichini E, Pizzichini MM, Efthimiadis A, Evans S, Morris MM, Squillace D (1996). Indices of airway inflammation in induced sputum: reproducibility and validity of cell and fluid-phase measurements.. Am J Respir Crit Care Med..

[9] Efthimiadis A, Pizzichini MM, Pizzichini E, Dolovich J, Hargreave FE (1997). Induced sputum cell and fluid-phase indices of inflammation: comparison of treatment with dithiothreitol vs phosphate-buffered saline.. Eur Respir J..

[10] Brightling CE, Monteiro W, Ward R, Parker D, Morgan MD, Wardlaw AJ (2000). Sputum eosinophilia and short-term response to prednisolone in chronic obstructive pulmonary disease: a randomised controlled trial.. Lancet..

[11] Belda J, Leigh R, Parameswaran K, O'Byrne PM, Sears MR, Hargreave FE (2000). Induced sputum cell counts in healthy adults.. Am J Respir Crit Care Med..

[12] Spanevello A, Confalonieri M, Sulotto F, Romano F, Balzano G, Migliori GB (2000). Induced sputum cellularity.. Reference values and distribution in normal volunteers. Am J Respir Crit Care Med..

[13] Davidson WJ, The S, Leigh R (2013). Establishing a normal range for induced sputum cell counts in Western Canada.. Can Respir J..

[14] Luo W, Chen Q, Chen R, Xie Y, Wang H, Lai K (2018). Reference value of induced sputum cell counts and its relationship with age in healthy adults in Guangzhou, Southern China.. Clin Respir J..

[15] Kim MY, Jo EJ, Lee SE, Lee SY, Song WJ, Kim TW (2014). Reference ranges for induced sputum eosinophil counts in Korean adult population.. Asia Pac Allergy..

[16] Veras TN, Pizzichini E, Steidle LJ, Rocha CC, Moritz P, Pizzichini MM (2011). Cellular composition of induced sputum in healthy adults.. J Bras Pneumol..

[17] Celli BR, MacNee W, ATS/ERS Task Force. (2004). Standards for the diagnosis and treatment of patients with COPD: a summary of the ATS/ERS position paper.. Eur Respir J..

[18] BTS guidelines for the management of chronic obstructive pulmonary disease. (1997). The COPD Guidelines Group of the Standards of Care Committee of the BTS.. Thorax..

[19] Westerhof GA, Korevaar DA, Amelink M, de Nijs SB, de Groot JC, Wang J (2015). Biomarkers to identify sputum eosinophilia in different adult asthma phenotypes.. Eur Respir J..

